# Co-ensiling garlic stalk with citrus pulp improves the fermentation quality and feed-nutritional value

**DOI:** 10.5713/ajas.19.0464

**Published:** 2019-08-26

**Authors:** Youn Hee Lee, Farhad Ahmadi, Young Il Kim, Young-Kyoon Oh, Wan Sup Kwak

**Affiliations:** 1 Division of Food Bio-science, College of Medical Life Sciences, Konkuk University, Chungju 27478, Korea; 2 Animal Nutrition and Physiology Team, National Institute of Animal Science, Rural Development Administration, Wanju 55365, Korea

**Keywords:** Garlic Stalk, Citrus Pulp, Ensiling, Lactic Acid Bacteria, Molasses, Feed

## Abstract

**Objective:**

Ensiling is a simple and effective method for long-term preservation; however, less information exists about the ensilability characteristics of garlic stalk (GS). Therefore, the objectives were to examine the ensiling feasibility of GS.

**Methods:**

The GS was ensiled alone or inoculated with *Lactobacillus plantarum* KU5 in the presence or absence of 5% molasses and ensiled for 7, 14, and 28 d. As an alternative storage method, GS was co-ensiled with wet citrus pulp (CP) at different proportions (GS:CP: 70:30, 60:40, 50:50, and 40:60). Analysis was made on physicochemical, fermentative, and nutritional parameters.

**Results:**

The GS was found to be a biomass which is difficult to ensile. A combination of microbial inoculant and molasses was successful in the improvement of the silage fermentation quality of GS. Co-ensiling of GS with wet CP at the mixing ratio of 50:50 provided the most desirable silage fermentation parameters, including the substantial lactic acid formation, low final pH, minor effluent loss, and the more favorable organoleptic properties.

**Conclusion:**

Co-ensiling GS with CP appears to be a simple and viable method of conservation, enabling the more efficient utilization of these by-product resources over a prolonged period.

## INTRODUCTION

The worldwide production of garlic amounted to 27 million tons in 2016, with China, India, and Korea being the major garlic producing countries [1]. Nearly 70% of the total garlic product is discarded as stalk, which could be used as a potential roughage source in a ruminant diet [2,3]. However, during the harvesting season large quantities of garlic stalk (GS) accumulate, which exceeds the amount needed for immediate use. Thus, the surplus is simply decomposed into the soil or incinerated, the latter resulting in environmental concerns [2,4]. This demands the development of an efficient conservation technology for its long-term use.

The ensiling process is an effective and simple conservation technology that is known to acidify biomass, thus it inhibits the growth of spoilage-causing microorganisms, thereby providing safe and long-term conservation for moist forages [5]. Inoculation with lactic acid bacteria (LAB) or supplementation with readily fermentable carbohydrates (readily metabolizable by LAB), such as molasses, proved to be successful in enhancing the efficiency of the ensiling process. This supplies an additional source of simple sugars for the growth and multiplication of LAB, thereby accelerating the silage acidification and avoiding microbial deterioration, especially when the ensiling biomass has a low concentration of soluble carbohydrates [5,6]. More recently, the simultaneous application of the LAB inoculant and molasses successfully improved the silage fermentation quality of various crops [7,8].

Citrus is one of the major fruits in Korea, which is mainly cultivated on Jeju Island and the southern coast line of the main land, with an estimated production of 0.68 million tons in 2011 [9]. An estimated 30% of citrus fruits is directed into the processing industries, resulting in the generation of about 50% to 60% organic waste [10]. Citrus pulp (CP), the main by-product of citrus processing facilities, is rich in the fermentable carbohydrates but high in moisture content (approximately 80% to 90%), which is associated with difficulties in its transportation, handling, and perishability [11,12]. Moreover, the high moisture content of CP results in effluent loss during ensiling, which is associated with the loss of silage nutrients and environmental worries arising from the pollution of surface and ground water [13]. Additionally, the high residual sugars give CP a wet and sticky nature that raises issues associated with its conservation in silos [12]. Earlier studies mainly investigated the dried CP as animal feed; however, drying is an expensive process and therefore an improper method for large-scale recycling of CP [12]. However, the appropriate mixing of CP with dry ingredients, to maintain the proper moisture content for silage making, would contribute to the efficient recycling of high-moisture CP [11]. Therefore, we hypothesized that combining GS (as a drier by-product) and CP could enhance the silage fermentation quality of the two by-products.

To our knowledge, limited reports are available concern ing the silage fermentation characteristics of GS. Therefore, the first objective was to evaluate how *Lactobacillus plantarum* (*L. plantarum*) inoculation without or with molasses would affect the silage fermentation patterns of GS. The second objective was to develop an alternative storage method through co-ensiling of GS with CP at different proportions, and determine the appropriate mixing ratio for efficient silage fermentation.

## MATERIALS AND METHODS

### Collection of garlic stalk and inoculum preparation

Different batches of sun-dried GS were provided from a garlic processing plant located in Uysung County, Kyung-Buk province and an agricultural marketing center located in Chungju city, Chung-Buk province in Korea. Before silage making, GS was cut into pieces of 20 to 30 mm and hand-mixed thoroughly, then divided into the respective treatments. *L. plantarum* KU5 (Accession No. HQ542227) inoculant was re-cultivated for 3 consecutive days on MRS (de Mann Rogosa Sharpe) broth (Difco Laboratories Inc., Detroit, MI, USA) at 36°C for 24 h, before being applied onto the biomass.

### Silage making

The GS was ensiled in nylon-polyethylene pouches (5 replicates per each treatment) without inoculant or with *L. plantarum* KU5 (LAB) in the absence or presence of % molasses (LAB+ M). Ensiling duration was 7, 14, and 28 d at room temperature (20°C to 24°C). The detailed description of the silage making procedure is reported in our previous article [8]. The suspension containing the LAB inoculant was applied onto the mixture using a manual sprayer at a rate to provide 1×10^6^ cfu/g of fresh mass. In a subsequent experiment, GS was mixed with CP at decremental proportions of 70:30, 60:40, 50:50, and 40:60 (wet weight), mixed manually, and then assigned to respective treatments. The packing density of approximately 200 kg dry matter (DM)/m^3^ was applied for all silages, before the sealing of the polyethylene silos. From the designated silo openings, the representative samples were collected randomly for the subsequent analyses.

### Chemical and microbiological analyses

Contents of DM, crude protein (N×6.25), ether extract, crude ash, neutral-detergent fiber (NDF), and acid detergent fiber, both corrected for residual ash, were determined according to the standard methods of the Association of Official Analytical Chemists [14]. True protein was determined after precipitation in 5% trichloroacetic acid solution [15]. Non-protein nitrogen was calculated as “100 − true protein”. Non-fibrous carbohydrate (NFC) was computed as 100 − (crude protein + NDF + ether extract + crude ash). For silage juice extraction, a 20 g sample of fresh or silage biomass was mixed with deionized autoclaved water (200 mL) and blended (DIAX 900, Heidolph, Schwabach, Germany) for 1 min. The suspension was filtered through two layers of medical gauze, and was used for the measurement of pH (HI 9321, Hanna Instruments, Woonsocket, RI, USA), water-soluble carbohydrates (WSC, glucose equivalent) [16], lactic acid [17], and NH3-N [18] using a UV-visible spectrophotometer (S-1100, Scinco, Daejeon, Korea). The viable colonies of total bacteria and LAB were counted using the spread-plating method on plate count agar and MRS agar, respectively. Plates were incubated (JSGI-050T, Hanyang Scientific Equipment Co., Ltd., Seoul, Korea) at 36°C for 48 h.

### Data analysis

Data analyses were performed using the general linear model procedure (Statistix7, 2000). Treatment effects were evaluated with the analysis of variance in a completely randomized design. Each mini-silo was considered as the experimental unit. Microbial counts were transformed to log10-scale before statistical analysis. The difference among treatments was identified using Tukey’s multiple range test. Values less than 0.05 probability level were considered significant. Tendency was discussed at 0.05<p<0.1.

## RESULTS AND DISCUSSION

### Silage fermentation quality of garlic stalk

The silage fermentation metabolites and chemical composition of GS ensiled for 7, 14, or 28 d, are presented in Table 1. Initial pH before ensiling was 8.06, which declined (p<0.01) as the length of ensiling prolonged (a 1.26-unit decline by d 28 of ensiling). Lactic acid concentration was greatest after 28 d of fermentation. This is associated with the development of LAB which tended to increase (p = 0.07) as fermentation duration continued. With ensiling, WSC content decreased while NH_3_-N formation increased considerably (a 4.4-times increase after 28 d of ensiling), which indicates the microbial conversion of WSC into lactic acid and protein breakdown during ensiling, respectively [5,19]. The chemical composition of GS silage differed negligibly as ensiling duration progressed. Crude protein content declined slightly with ensiling (p< 0.002), which can possibly be explained by the protein breakdown (proteolysis) during ensiling, which releases NH_3_-N [5].

An approximate number of 8 log _10_ cfu of LAB per gram of fresh biomass is the required LAB number to ensure the fast decline of silage pH [20]. In the present experiment, although the population of epiphytic LAB was adequate (7.93 log_10_ cfu/g) to promote fast acidification and efficient ensiling, the silage fermentation was not successful, as evidenced by high NH_3_-N content and high silage pH after 28 d of fermentation. This suggests that GS is a difficult-to-ensile biomass. This can be justified by three explanations: i) The GS contains an inadequate content of WSC, which probably did not provide sufficient sugars for lactic acid production, and thus silage mass acidification [5,6]. ii) The GS contains a high ash concentration (approximately 15%), which would increase the buffering capacity, thus resisting silage mass acidification [5]. iii) GS has a hollow and tubular structure [4] that increases the porosity of GS mass, accelerates the aerobic microbial activity during the early phase of ensiling, and thus converts readily available carbohydrates into carbon dioxide and water [5]. These factors collectively result in an inefficient anaerobic fermentation and slow acidification [20]. Based on these assumptions, the follow-up experiment attempted to improve the fermentation quality of GS with LAB inoculation in the presence of molasses as a source of WSC.

The silage fermentation dynamics of GS inoculated with LAB in the absence or presence of molasses in relation to the length of ensiling (7, 14, and 28 d), are presented in Table 2. Generally, the fermentation quality parameters of GS silage treated with a combination of LAB+M were preferable to those of untreated or LAB-treated silages. After 7 d, silage pH declined from the initial value of 7.7 in the control silage to 5.8 in the LAB+M silage, indicating that the combination of LAB and molasses promoted the favorable silage fermentation pattern.

Inoculation with LAB prior to ensiling has been successful in the promotion of desirable fermentation patterns. However, when the epiphytic population of LAB exceeds the inoculant application rate, the domination of inoculant bacteria in the silage is difficult [21]. In the present experiment, the natural population of LAB in GS was close to 8 log cfu/g of fresh GS (prior to ensiling), which considerably exceeded the LAB application rate (10^6^ cfu/g of fresh GS). Surprisingly, LAB+M treatment promoted the more favorable fermentative patterns (with respect to a lower pH and less NH_3_-N production) than untreated or LAB-treated silage. After 28 d of ensiling, LAB+M-treated silage showed a 35% increase in lactic acid content and a 0.9-unit decline in pH, with respect to the LAB-inoculated silage. In support, Huisden et al [19] reported that the addition of molasses to corn silage supplied an extra source of WSC for LAB metabolism, which possibly allowed their domination in the microbial community of the silage, thereby stimulating favorable silage fermentation patterns.

Earlier findings confirmed that the combined use of the LAB inoculant and molasses improved the silage quality indices of various crops [7,8,22]. For example, our recent investigation [8] found that the combination of LAB and 5% molasses efficiently improved the silage quality parameters of spent mushroom substrate in both laboratory-scale and ton-bag silos. Presently, when GS was ensiled without molasses or a microbial inoculant, silage pH slightly declined (6.80 after 28 d of ensiling), which was accompanied by the high NH_3_-N accumulation (631 ppm). However, when GS was ensiled with LAB+M, the silage pH dropped to 5.3 (after 28 d of ensiling) and as anticipated, less NH_3_-N was formed (Table 2). This observation was expected, because when silage pH declines more quickly, excessive proteolysis is suppressed during ensiling, which contributes to less NH_3_-N formation [5].

The chemical composition of GS ensiled with LAB in the absence or presence of molasses in relation to the length of ensiling, is presented in Table 3. No difference in the chemical composition was seen after 7 d of ensiling. However, as ensiling duration prolonged, the NDF concentration decreased in LAB+M-treated silage, which is possibly associated with the hydrolysis of cell wall fractions that are more digestible during ensiling fermentation [19].

### Co-ensiling of garlic stalk with citrus pulp

This experiment evaluated the co-ensiling of GS with CP as an alternative storage technology, which has been successful in improving the silage fermentation quality of several feedstuffs [23]. For example, the recent co-ensiling of wheat straw with sugar beet waste has proved to be a successful storage method for their long-term preservation [24]. The typical silage quality parameters and chemical composition of GS and CP, ensiled alone or together (GS 70%+CP 30%) after 7, 14, or 28 d of ensiling, are reported in Tables 4 and 5, respectively. Generally, GS ensiled with CP exhibited more desirable fermentation characteristics in comparison to GS silage alone. For example, after 14 d of ensiling, the GS 70%+CP 30% silage exhibited a 56% increase in lactic acid content which represented a 0.4-unit decline in silage pH, with respect to GS ensiled alone. The nutrient composition of GS 70%+CP 30% silage was comparable to that of GS ensiled alone. However, after 14 and 28 d of ensiling, a greater NFC concentration was recorded for GS 70%+CP 30% silage, which is associated with an NDF content that diminished as ensiling time continued. For example, after 28 d of ensiling, NDF concentration decreased by 7.5% with respect to GS silage, which was possibly caused by the dissolution of the more digestible NDF fractions during ensiling [19]. These findings showed promise in the possibility of the successful preservation of GS when co-ensiled with CP, leading to the follow-up experiments to determine the proper mixing level of GS and CP for efficient silage fermentation.

**Figure 1:**
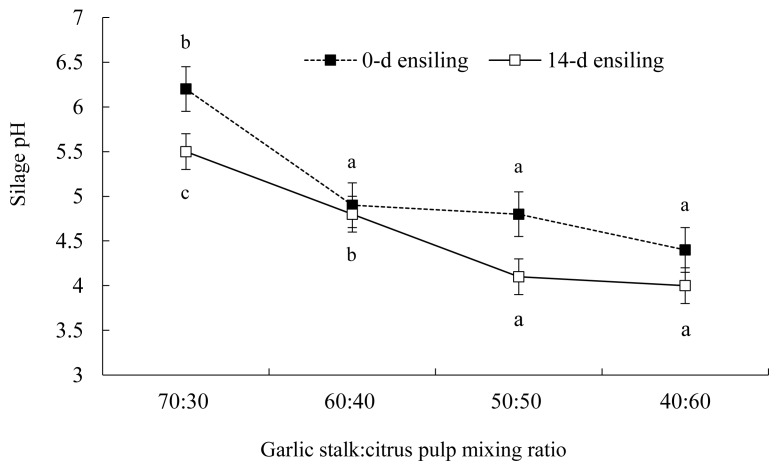
Changes in silage pH of garlic stalk co-ensiled with citrus pulp at varying proportions. ■, 0 d of ensiling; □, after 14 d of ensiling. ^a–c^ Means with different letters within the same line differ (p<0.05). Error bars at each point represent standard error.

**Figure 2:**
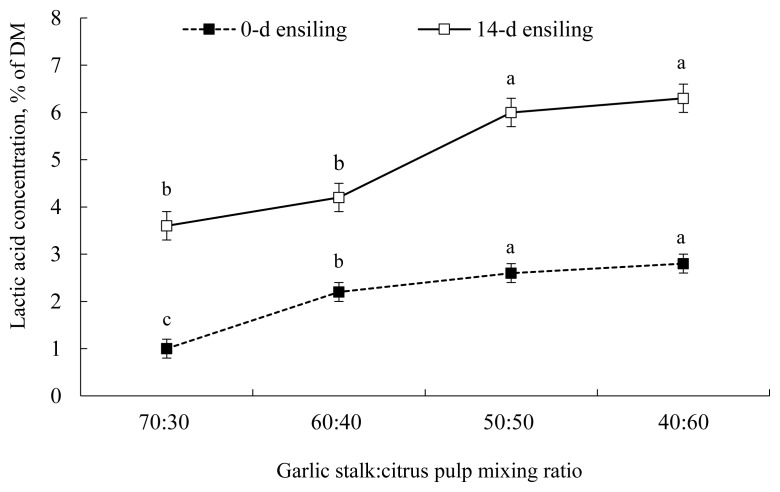
Lactic acid concentration of garlic stalk co-ensiled with citrus pulp at varying proportions. ■, 0 d of ensiling; □, after 14 d of ensiling. ^a–c^ Means with different letters within the same line differ (p<0.05). Error bars at each point represent standard error.

The pH of GS co-ensiled with incremental proportions of CP is illustrated in Figure 1. After 14 d of ensiling, the pH of GS 50%+CP 50% silage declined markedly, which is suggestive of successful silage fermentation that is known to slow down or inhibit the growth of fungi, molds, and yeasts [25]. Consistent with our findings, Migwi et al [23] reported that when 5% molasses was added to the mixture of wheat straw+ broiler litter, no effect on silage pH was detected. However, when the proportion of CP increased from 0 to 30% in the silage, the pH declined markedly [23]. Similarly, when CP was ensiled alone, lactic acid concentration was negligible (1.02% of DM, after 14 d of ensiling; Figure 2). However, when CP was co-ensiled with GS at a 50:50 proportion, lactic acid production increased markedly (6.0% of DM, after 14 d of ensiling).

The silage fermentation, sensory, and physical parame ters of GS co-ensiled with incremental proportions of CP are shown in Table 6. After 2 weeks of fermentation, despite the substantial increase in lactic acid production with increasing CP proportion (Figure 2), total viable colonies of LAB remained unaffected with increasing proportions of CP in the silage. This might be explained by the counting technique used, as this requires actively growing cells. It has been proposed that many LAB exist on the biomass surfaces that are viable but not culturable by using the traditional plating methods [26]. Assuming this proposition, it appears that the increased availability of metabolic water provided by the increased proportion of CP into GS biomass supported the higher metabolic activity of the microbial ecology of silage, particularly LAB [27]. In agreement, Muck [28] ensiled alfalfa at different DM levels (17% to 73%) and found that the greatest levels of lactic acid were produced in silages of 40% to 55% DM. Likewise, Mthiyane et al [29] found that when water was added to sugarcane tops silage, lactic acid production was restricted, which was explained by the multiplication and domination of heterofermentative LAB in high-moisture silage, as this generates the mixed metabolites of acetic acid, ethanol, and lactic acid. Essentially, silage making at the optimal moisture level should provide enough moisture for LAB growth and metabolism to decrease pH and prevent the growth of putrefactive microorganisms, while also providing enough dryness to minimize effluent production [5,13]. Presently, it appears that the 50:50 mixing proportion provided the appropriate moisture level that favored the metabolic activity of LAB in the mixed silage of GS and CP, and thus promoted substantial lactic acid production. However, the reasons why lactic acid production increased with moisture alterations of the silage are still not clear and necessitate more studies to identify the contributory factors to this observation.

As the GS:CP mixing proportion decreased, NH _3_-N concentration lessened, which is indicative of diminished proteolysis and deamination during ensiling [5]. After 14 d of ensiling, NH_3_-N content accounted for 25.4% of the total N in GS 50% +CP 50% silage, which is approximately 70% less than the silages with 30% or 40% CP. The fast acidification of silage mass suppresses the growth of putrefying microorganisms, this probably occurred as the mixing ratio of GS:CP decreased, resulting in a lower formation of NH_3_-N, which is associated with the improved utilization of silage-N and thus microbial protein synthesis in the rumen [20]. Decreasing the porosity of forage silage mass using supplementary feed sources, such as corn meal, with fine particle sizes may accelerate the silage pH decline, diminish inefficient plant respiration (less heat generation), and thus promote effective anaerobic fermentation [30]. This series of events is known to minimize proteolysis, which is accelerated at high silage pH and temperature [20]. It appears that the co-mixing of CP and GS decreased the porosity of the GS mass, which thereby promoted efficient silage fermentation that minimized NH_3_-N formation.

No putrid odor or moldy spots were detected in the ex perimental silages (Table 6), which suggests that the silages underwent a successful fermentation. However, fermentation odor increased and garlic odor decreased as the incremental levels of CP were incorporated into GS silage. The absorption degree was highest when 40% or 50% CP was incorporated into GS, which indicates that the effluent lost from CP was efficiently assimilated into GS biomass. This finding is of great importance as CP has a high moisture content and its ensiling alone would cause problems associated with effluent and silage nutrient losses [12], which can be minimized when co-ensiled with GS at the proper mixing ratio. Overall, this experiment found that when GS and CP were combined at a 50:50 proportion, the best ensiling and physical properties were achieved, as demonstrated by the domination of LAB, low final pH, substantial lactic acid formation, and negligible effluent loss, thus opening an avenue for the simultaneous and long-term conservation of these waste residues.

The chemical composition of GS co-ensiled with incremen tal proportions of CP is shown in Table 7. As the proportion of CP increased in the silage mass the DM, NDF, and ether extract contents declined, and NFC content increased as expected. After 14 d of ensiling, GS 50%+CP 50% silage resulted in a 6.9% reduction in NDF content, with respect to GS ensiled with 30% CP, resulting in a higher NFC concentration, which is suggestive of the improved feed-nutritional quality of the mixed silage of GS and CP for ruminant feed.

## CONCLUSION

A huge amount of GS is generated during the harvesting season, an amount considerably exceeding the immediate use. This surplus is mainly composted into soil or incinerated, which is associated with serious environmental concerns. The simultaneous application of *L. plantarum* inoculant and molasses favorably promoted the silage fermentation quality of GS. As an alternate storage method, the effective silage fermentation was also achieved when GS was co-ensiled with CP at the mixing ratio of 50:50. Co-ensiling of GS with CP appears to be a simple and feasible method of preservation, which would provide the more efficient utilization of these waste resources over longer periods, especially in places where they are generated in large quantities.

## Figures and Tables

**Table 1:** Silage quality parameters and chemical composition of garlic stalk in relation to the ensiling duration

Items	Ensiling duration (d)				SE	p-value

	0	7	14	28		
Silage quality parameters						
pH	8.06^c^	7.68^b^	7.69^b^	6.80^a^	0.28	<0.001
WSC (% of DM)	2.10^a^	1.51^b^	1.92^a^	1.44^b^	0.20	0.021
Lactic acid (% of DM)	0.42^a^	0.40^a^	0.42^a^	0.49^b^	0.03	0.013
NH_3_-N (ppm)	143^a^	311^b^	424^b^	631^c^	24.8	<0.001
Lactic acid bacteria, log_10_ cfu/g1)	7.93	8.36	8.15	8.23	0.13	0.069
Chemical composition						
Dry matter (%)	63.2	64.4	63.5	62.8	0.81	0.325
Crude protein (% of DM)	7.02^a^	7.01^a^	6.40^b^	6.32^b^	0.20	0.002
True protein (% of crude protein)	57.8	52.8	56.2	52.8	1.31	0.123
NPN (% of crude protein)	42.2	47.2	43.8	47.2	1.33	0.123
NDF (% of DM)	49.7	50.4	48.8	49.4	1.41	0.532
ADF (% of DM)	48.4	49.9	50.2	48.9	1.54	0.806
Ether extract (% of DM)	1.73	1.58	1.57	1.70	0.21	0.974
Crude ash (% of DM)	15.9	15.5	16.9	16.7	0.34	0.322

Means of five observations.

SE, standard error; WSC, water-soluble carbohydrates; DM, dry matter; NPN, non-protein nitrogen; NDF, neutral detergent fiber; ADF, acid detergent fiber.

1) Colony-forming units per gram of fresh biomass or silage mass.

a–c Means with different superscripts within the same row differ.

**Table 2:** Effects of microbial inoculant without or with molasses on the silage quality parameters of garlic stalk after 7, 14, and 28 d of ensiling

Items	Treatment1)			SE	p-value

	Control	LAB	LAB+M		
Ensiled for 7 d					
pH	7.7^a^	7.5^a^	5.8^b^	0.31	0.001
WSC (% of DM)	1.5^c^	2.0^b^	2.4^a^	0.23	0.002
Lactic acid (% of DM)	0.40	0.42	0.44	0.02	0.298
NH_3_-N (ppm)	311	349	284	27.9	0.116
Lactic acid bacteria, log_10_ cfu/g2)	8.4	8.8	8.8	0.20	0.107
Ensiled for 14 d					
pH	7.7^a^	7.7^a^	5.4^b^	0.30	<0.001
WSC (% of DM)	1.9^ab^	1.7^b^	2.3^a^	0.21	0.009
Lactic acid (% of DM)	0.42^b^	0.42^b^	0.64^a^	0.07	0.027
NH_3_-N (ppm)	424^ab^	502^a^	362^b^	30.9	0.006
Lactic acid bacteria, log_10_ cfu/g	8.2^b^	8.4^b^	8.9^a^	0.11	0.001
Ensiled for 28 d					
pH	6.8^a^	6.3^ab^	5.3^b^	0.31	0.042
WSC (% of DM)	1.4	1.4	1.7	0.10	0.050
Lactic acid (% of DM)	0.49	0.54	0.66	0.06	0.070
NH_3_-N (ppm)	631	553	475	56.9	0.071
Lactic acid bacteria, log_10_ cfu/g	8.2	8.4	8.5	0.24	0.230

Means of five observations.

SE, standard error; DM, dry matter; WSC, water-soluble carbohydrates.

1) Control, no inoculant; LAB, 0.5% (v/w) *Lactobacillus plantarum* KU5; LAB+M, 0.5% (v/w) *Lactobacillus plantarum* KU5+5% molasses.

2) Colony-forming units per gram of fresh biomass or silage mass.

a–c Means with different superscripts within the same row differ.

**Table 3:** Effects of microbial inoculant without or with molasses on the chemical composition of garlic stalk after 7, 14, and 28 d of ensiling

Items	Treatment1)			SE	p-value

	Control	LAB	LAB+M		
Ensiled for 7 d					
Dry matter (%)	64.4	63.3	63.9	0.73	0.356
Crude protein	7.0	7.1	7.0	0.31	0.868
True protein (% of crude protein)	52.8	53.4	52.2	1.60	0.751
NPN (% of crude protein)	47.2	46.6	47.8	1.61	0.751
Crude ash	15.5	15.4	15.2	0.32	0.666
NDF	50.4	51.7	51.4	1.30	0.603
ADF	49.9	49.7	48.4	1.21	0.448
Ensiled for 14 d					
Dry matter (%)	63.5	63.1	61.5	1.14	0.191
Crude protein	6.4^b^	6.6^b^	7.3^a^	0.20	0.012
True protein (% of crude protein)	56.2^a^	52.2^ab^	48.4^b^	1.71	0.004
NPN (% of crude protein)	43.8^b^	47.8^ab^	51.6^a^	1.71	0.004
Crude ash	16.9	16.1	16.7	0.81	0.560
NDF	48.8^ab^	49.9^a^	46.1^b^	1.33	0.036
ADF	50.2	48.9	47.7	1.31	0.194
Ensiled for 28 d					
Dry matter (%)	62.8	61.2	63.1	0.83	0.067
Crude protein	6.3	6.8	6.7	0.21	0.150
True protein (% of crude protein)	52.8	51.4	52.2	1.81	0.748
NPN (% of crude protein)	47.2	48.6	47.8	1.84	0.748
Crude ash	16.7^a^	16.1^a^	15.2^b^	0.33	0.003
NDF	49.4^a^	48.4^a^	46.6^b^	0.40	<0.001
ADF	48.0	46.1	46.0	0.81	0.052

Means of five observations.

SE, standard error; NPN, non-protein nitrogen; NDF, neutral detergent fiber; ADF, acid detergent fiber.

1) Control, no inoculant; LAB, 0.5% (v/w) *Lactobacillus plantarum* KU5; LAB+M, 0.5% (v/w) *Lactobacillus plantarum* KU5+5% molasses.

Values are based on % of DM, unless stated.

a,b Means with different superscripts within the same row differ.

**Table 4:** Effects of the co-ensiling of garlic stalk with citrus pulp on the silage quality parameters after 7, 14, and 28 d of ensiling

Items	Treatment1)			SE	p-value

	GS 100%	CP 100%	GS 70% +CP 30%		
d 0 (before ensiling)					
pH	7.5^a^	3.2^c^	5.7^b^	0.11	<0.001
Lactic acid (% of DM)	0.16^b^	2.45^a^	0.19^b^	0.09	<0.001
Ensiled for 7 d					
pH	6.6^a^	3.0^c^	5.6^b^	0.10	<0.001
Lactic acid (% of DM)	0.17^b^	1.84^a^	0.20^b^	0.10	<0.001
Ensiled for 14 d					
pH	6.3^a^	3.1^c^	5.9^b^	0.11	<0.001
Lactic acid (% of DM)	0.16^b^	1.02^a^	0.25^b^	0.10	<0.001
Ensiled for 28 d					
pH	5.7^a^	3.1^b^	5.4^a^	0.10	<0.001
Lactic acid (% of DM)	0.18^b^	1.12^a^	0.26^b^	0.08	<0.001

Means of five observations.

SE, standard error; DM, dry matter.

1) GS, garlic stalk; CP, citrus pulp.

a–c Means with different superscripts within the same row differ.

**Table 5:** Effects of the co-ensiling of garlic stalk with citrus pulp on the chemical composition after 7, 14, and 28 d of ensiling

Items	Treatment1)			SE	p-value

	GS 100%	CP 100%	GS 70% +CP 30%		
Ensiled for 0 d					
Dry matter (%)	61.1^b^	13.7^c^	63.4^a^	0.31	<0.001
Crude protein	5.4^b^	9.3^a^	5.5^b^	0.12	<0.001
NDF	52.1^a^	24.0^b^	51.5^a^	2.22	<0.001
Ether extract	1.8^b^	4.2^a^	2.1^b^	0.30	<0.001
Crude ash	13.4^a^	4.1^b^	12.8^a^	0.43	<0.001
Ensiled for 7 d					
Dry matter (%)	62.0^a^	13.4^b^	63.0^a^	0.64	<0.001
Crude protein	5.3^b^	9.4^a^	5.4^b^	0.26	<0.001
NDF	52.5^a^	27.3^b^	52.5^a^	2.61	<0.001
Ether extract	1.7^b^	2.5^a^	1.9^b^	0.11	<0.001
Crude ash	14.4^a^	4.1^c^	12.8^b^	0.40	<0.001
Ensiled for 14 d					
Dry matter (%)	63.0^b^	13.7^c^	64.8^a^	0.51	<0.001
Crude protein	5.1^b^	9.1^a^	5.5^b^	0.23	<0.001
NDF	56.6^a^	26.7^c^	43.4^b^	1.04	<0.001
Ether extract	1.7^b^	3.4^a^	2.1^b^	0.21	<0.001
Crude ash	12.9^a^	4.0^b^	12.6^a^	0.32	<0.001
Ensiled for 28 d					
Dry matter (%)	61.5^b^	14.0^c^	62.8^a^	0.34	<0.001
Crude protein	5.2^b^	9.2^a^	5.6^b^	0.25	<0.001
NDF	54.1^a^	24.6^c^	46.5^b^	1.30	<0.001
Ether extract	1.9^b^	3.9^a^	2.1^b^	0.12	<0.001
Crude ash	13.3^a^	4.0^b^	13.0^a^	0.21	<0.001

Means of five observations.

SE, standard error; NDF, neutral detergent fiber.

1) GS, garlic stalk 100%; CP, citrus pulp 100%; GS+CP, garlic stalk 70%+citrus pulp 30%.

Values are based on % of DM, unless stated.

a–c Means with different superscripts within the same row differ.

**Table 6:** Effects of the addition of citrus pulp to garlic stalk on the silage quality, sensory, and physical parameters after 14 d of ensiling

Items	Treatment1)				SE	p-value

	GS 70%+CP 30%	GS 60%+CP 40%	GS 50%+CP 50%	GS 40%+CP 60%		
d 0 (before ensiling)						
WSC (of DM)	1.7	1.8	1.9	1.7	0.12	0.233
NH_3_-N (ppm)	291^b^	395^a^	279^b^	248^b^	29.1	<0.001
Ensiled for 14 d						
WSC (% of DM)	1.7	1.9	1.9	1.8	0.14	0.418
NH_3_-N (ppm)	444^ab^	533^a^	325^bc^	279^c^	36.7	<0.001
Lactic acid bacteria, log_10_ cfu/g2)	7.3	7.2	7.7	7.1	0.20	0.101
Sensory and physical parameters						
Fermentation odor3)	3.0^b^	3.1^b^	3.4^a^	3.4^a^	0.10	<0.001
Putrid odor4)	0	0	0	0	-	-
Garlic odor5)	1.0^a^	0.6^b^	0.4^c^	0.3^c^	0.11	<0.001
Moldy appearance6)	0	0	0	0	-	-
Absorption degree7)	3.6^b^	4.0^a^	4.2^a^	3.4^b^	0.12	<0.001

Means of five observations.

SE, standard error; WSC, water-soluble carbohydrates; DM, dry matter.

1) GS, garlic stalk; CP, citrus pulp.

2) Colony-forming units per gram of fresh biomass or silage mass.

3) Based on a 5-point scale as follows: 1 = very bad, 2 = bad, 3 = moderate, 4 = good, 5 = very good.

4) Based on a 2-point scale as follows: 0 = no putrid odor, 1 = putrid odor.

5) Based on a 2-point scale as follows: 0 = no garlic odor, 1 = garlic odor.

6) Based on a 2-point scale as follows: 0 = no moldy appearance, 1 = moldy appearance.

7) Based on a 5-point scale as follows: 1 = negligible, 5 = very much.

a–c Means with different superscripts within the same row differ.

**Table 7:** Effects of the addition of incremental proportions of citrus pulp to garlic stalk on the chemical composition after 14 d of ensiling

Items	Treatment1)				SE	p-value

	GS 70%+CP 30%	GS 60%+CP 40%	GS 50%+CP 50%	GS 40%+CP 60%		
d-0 (before ensiling)						
Dry matter (%)	63.5^a^	57.4^b^	45.5^c^	44.5^c^	1.44	<0.001
Crude protein	7.7^ab^	7.8^a^	7.5^ab^	7.3^b^	0.10	0.014
NDF	41.5^b^	44.7^a^	37.5^c^	35.0^c^	1.01	<0.001
Acid detergent fiber	45.2^b^	49.4^a^	42.3^c^	43.2^bc^	1.04	<0.001
Ether extract	1.4^a^	1.1^b^	1.1^b^	1.2^b^	0.12	0.001
Crude ash	12.6^a^	10.2^c^	11.1^b^	11.7^ab^	0.31	<0.001
NFC	36.8^b^	36.2^b^	42.8^a^	44.8^a^	1.10	<0.001
Ensiled for 14 d						
Dry matter (%)	62.9^a^	56.3^b^	48.3^c^	42.2^d^	2.14	<0.001
Crude protein	7.8^b^	9.3^a^	8.0^b^	7.9^b^	0.15	<0.001
NDF	44.3^a^	45.3^a^	37.4^b^	32.9^c^	0.50	<0.001
Acid detergent fiber	44.7^ab^	46.9^a^	43.1^b^	38.8^c^	1.03	<0.001
Ether extract	1.7^b^	2.0^a^	2.1^a^	2.1^a^	0.11	0.004
Crude ash	12.4	12.6	12.5	11.8	0.34	0.167
NFC	33.8^c^	30.8^d^	40.0^b^	45.3^a^	0.63	<0.001

Means of five observations.

SE, standard error; NDF, neutral detergent fiber; NFC, non-fibrous carbohydrates; DM, dry matter.

1) GS, garlic stalk; CP, citrus pulp.

Values are based on % of DM, unless stated.

a–d Means with different superscripts within the same row differ.
